# Return to work in prostate cancer survivors – findings from a prospective study on occupational reintegration following a cancer rehabilitation program

**DOI:** 10.1186/s12885-018-4614-0

**Published:** 2018-07-20

**Authors:** Anneke Ullrich, Hilke Maria Rath, Ullrich Otto, Christa Kerschgens, Martin Raida, Christa Hagen-Aukamp, Corinna Bergelt

**Affiliations:** 1 Department of Medical Psychology, University Medical Center Hamburg-Eppendorf, Center for Psychosocial Medicine, Martinistrasse 52, 20246 Hamburg, Germany; 2 Rehabilitation Clinics Hartenstein GmbH, Clinic Quellental, Bad Wildungen, Germany; 3 Vivantes Rehabilitation Clinic GmbH, Berlin, Germany; 4 HELIOS Rehabilitation Clinic Bergisch-Land, Wuppertal, Germany; 5 Niederrhein Rehabilitation Clinic, Korschenbroich, Germany

**Keywords:** Prostate cancer, Oncology, Return to work, Time until return to work, Rehabilitation, Psycho-oncology, Predictor

## Abstract

**Background:**

This prospective multicentre-study aimed to analyze return to work (RTW) among prostate cancer survivors 12 months after having attended a cancer rehabilitation program and to identify risk factors for no and late RTW.

**Methods:**

Seven hundred eleven employed prostate cancer survivors treated with radical prostatectomy completed validated self-rating questionnaires at the beginning, the end, and 12 months post rehabilitation. Disease-related data was obtained from physicians and medical records. Work status and time until RTW were assessed at 12-months follow-up. Data were analyzed by univariate analyses (t-tests, chi-square-tests) and multivariate logistic regression models (OR with 95% CI).

**Results:**

The RTW rate at 12-months follow-up was 87% and the median time until RTW was 56 days. Univariate analyses revealed significant group differences in baseline personal characteristics and health status, psychosocial well-being and work-related factors between survivors who had vs. had not returned to work. Patients’ perceptions of not being able to work (OR 3.671) and feeling incapable to return to the former job (OR 3.162) were the strongest predictors for not having returned to work at 12-months follow-up. Being diagnosed with UICC tumor stage III (OR 2.946) and patients’ perceptions of not being able to work (OR 4.502) were the strongest predictors for late RTW (≥ 8 weeks).

**Conclusions:**

A high proportion of prostate cancer survivors return to work after a cancer rehabilitation program. However, results indicate the necessity to early identify survivors with low RTW motivation and unfavorable work-related perceptions who may benefit from intensified occupational support during cancer rehabilitation.

**Electronic supplementary material:**

The online version of this article (10.1186/s12885-018-4614-0) contains supplementary material, which is available to authorized users.

## Background

Return to work (RTW) is highly relevant for cancer recovery and the social reintegration of working-age cancer patients, as work provides social connections, self-esteem and independence, and helps to regain a sense of normalcy [1, 2]. Not returning to work after cancer presents a challenge for both the individual and the society as a whole [3, 4]. An international review reporting a mean RTW rate of 63.5% indicates that approximately one third of cancer patients do not work 1 year after diagnosis [5]. As some adverse effects of not working may increase with the time passing, time until RTW is a relevant outcome of successful occupational reintegration [4]. For example, long-term sickness absence has been shown to increase the risk of early retirement [6]. A growing body of evidence suggests personal, disease- and treatment-related, psychosocial and work-related factors that may be barriers for RTW or may cause delayed RTW [4–11].

However, surprisingly little research has focused on RTW outcomes in survivors of prostate cancer, although it is the most common malignancy among men in economically developed countries [12]. In Europe, in 2012 approximately 119,000 men of working age were newly diagnosed with prostate cancer [13]. As different cancer sites are associated with varying prognosis, symptom burden and treatment procedures, RTW research should be geared to specific cancer survivor groups. Further, work should be considered as a key aspect of life and self-identity among working-age men [14–16], and studies on cancer and employment suggest gender-differences regarding various RTW outcomes [17]. In prior studies, prostate cancer survivors showed lower employment rates [7, 18], a higher probability to retire [19], longer absence from work [11, 20] and worse levels of work ability [21, 22] compared to men without cancer diagnosis. However, some studies indicate that prostate cancer survivors show better RTW outcomes, such as lower work disability rates [23] and the level of reduced employment participation [24], than survivors from other cancer entities.

In Germany, depending on criteria of rehabilitation need and prognosis, patients are entitled to participate in cancer rehabilitation programs following acute treatment, which are mainly provided in an inpatient setting and generally last 3 weeks [25]. According to the World Health Organization’s International Classification of Functioning, Disability and Health (ICF) [26], those programs aim to help patients regaining functioning, activity and participation through multimodal treatment concepts, with standard application of occupational counseling for working-age patients. For patients of working age, costs for such programs are most commonly covered by the German Pension Insurance Agency [27].

We conducted a study in a population of employed prostate cancer survivors who participated in a cancer rehabilitation program immediately following radical prostatectomy. The purpose of our study was (1) to analyze the RTW rate and time until RTW in this patient population 12 months after having attended the rehabilitation program and (2) to identify socio-demographic, disease-specific, psychosocial and work-related factors associated with not having returned to work and late RTW at 12-months follow-up. With the second aim, we sought to detect survivors at risk for adverse RTW outcomes at an early stage of the RTW process.

## Methods

### Study design and study population

In this prospective multicentre-study, survivors were consecutively enrolled in four German specialized rehabilitation clinics between October 2010 and June 2012. Eligible survivors were recruited during the initial clinical consultation at the beginning of the rehabilitation program. Survivors were included if they met the following criteria:

- localized prostate cancer (no evidence of lymphogenic and distant metastasis)
- starting the rehabilitation program within 14 days after the end of acute treatment (“post-acute rehabilitation”)
- working age (18–64 years)
- paid employment prior to radical prostatectomy
- written informed consent provided for study participation, data analysis and publication.

The exclusion criteria were the following:

- early retirement or having applied for a pension
- severe psychological or physical stress (physician’s assessment)
- inadequate knowledge of the German language.

The study protocol was approved by the ethics committee of the General Medical Council of Hamburg (PV3547) and the department of data security of the German Pension Insurance Agency.

Patient-reported data were collected by questionnaires at the beginning, at the end, and 12 months after the end of the rehabilitation program. The first two questionnaires were handed over by the treating physicians, the follow-up questionnaire was mailed to the respondents. Disease-specific data were given by physicians and retrieved from medical records.

### Rehabilitation programs

Based on guidelines concerning cancer rehabilitation, prostate cancer survivors received a (non study-specific) comprehensive multidisciplinary medical rehabilitation program with high treatment intensity. All rehabilitation clinics were certified for provision of prostate cancer rehabilitation programs. Three clinics provided rehabilitation for patients of different cancer types and one was a clinic for urological cancers. Clinics offered inpatient and/or fulltime outpatient cancer rehabilitation, with the National Association for Rehabilitation demanding comparable therapeutic treatment and staffing of the clinic for both rehabilitation settings [28]. Both in- and outpatient rehabilitation programs include medical treatment, physical training, psychological support/therapy, social counseling as well as patient education. Categories of therapeutic treatment are constituted in the Pension Insurance’s KTL classification system [29]. Actual provision of care might vary across patient groups. To collect information on rehabilitation processes in the studied cohort of prostate cancer survivors, kind and dose of treatments were derived from routine data and have been reported elsewhere [30]. Patients of both rehabilitation settings received a comparable treatment dose (approx. 12 h per week), but to some extent differed in the kind of treatments. Largest group differences were found in the category “sports and exercise therapy” for the benefit of outpatients and in the category “ergotherapy, occupational therapy and other functional therapies” for the benefit of inpatients. Discrepancies were due to differences regarding patients’ characteristics in the in- and outpatient setting.

### Measurements

#### Variables on RTW outcomes

Data regarding RTW rate and time until RTW were collected at 12-months follow-up. The current work status was assessed by confirmation of one of the following options: being employed part- or full-time, unemployed, disability or retirement pension. Survivors were either allocated to the group ‘having returned to work’ (working part- or full-time) or ‘not having returned to work’ (including the remaining categories) following a binary approach of RTW. Furthermore, survivors were asked to report on the exact date of their RTW following the rehabilitation program. The date of RTW was defined as time point when survivors started to work in any payed employment after the end of the rehabilitation program, independent of potential changes related to the working situation (e.g. reduced working hours, changes of working tasks or employer). Almost all survivors had returned to work without any changes of the job situation or weekly hours worked compared to the time prior to the prostate cancer diagnosis [31]. Time until RTW (in days) was calculated by linkage of the patient-reported date of RTW to the date of discharge from the rehabilitation clinics retrieved from medical records. The sample was dichotomized at the median time until RTW (8 weeks) and each survivor was assigned to the group ‘early RTW’ (< 8 weeks) or ‘late RTW’ (≥ 8 weeks).

#### Potential predictor variables

The set of potential predictors was chosen to fit the model on cancer and work as proposed by Feuerstein et al. [32] comprising seven dimensions associated with RTW outcomes: survivor’s personal characteristics, health status and well-being, function, symptoms, work demands, work environment, and healthcare system. We examined a comprehensive set of factors from each dimension by mainly using validated self-rating scales (German versions). All data were obtained at the beginning of the rehabilitation program (baseline).

Survivors reported on *personal characteristics* (date of birth, marital status; data collection about educational level, monthly household net income and occupational position adapted from the social class index by Winkler and Stolzenberg [33]). Data on *health status* (surgical method, UICC tumor stage [34], time since diagnosis via punch biopsy, Karnofsky performance status [35], extent of urinary incontinence, comorbidities) and *healthcare system* (rehabilitation setting) were provided by physicians or retrieved from medical records. Urinary incontinence was clinically assessed by physicians using a study-specific scale (‘°0: no leakage’, ‘°1: only in the afternoon’, ‘°2: already before noon’, ‘°3: also at night’).

*Well-being, function and symptoms* were assessed using the Hospital Anxiety and Depression Scale (HADS), the European Organization for Research and Treatment of Cancer Quality of Life Questionnaire (EORTC QLQ-C30) and its prostate cancer-specific module (-PR25). The HADS [36] was specifically designed to measure anxiety and depression in somatically ill patients. The instrument consists of two subscales for anxiety and depression, both ranging from 0 to 21 points, with cut-offs of ≥11 indicating clinically relevant symptom levels. The EORTC QLQ-C30 [37] measures health-related quality of life and consists of six functional (global health status; physical, role, social, emotional, cognitive functioning) and 15 symptom scales. The EORTC QLQ-PR25 [38] assesses sexual functioning and four symptom scales (urinary, bowel and hormonal treatment-related symptoms, bother due to use of incontinence aid). All scale scores are linearly transformed to a 0–100 scale, with higher scores reflecting either higher levels of functioning or higher symptom burden.

Factors of *work demands* and *work environment* were assessed using the Screening Instrument Work and Occupation (German Abbrev.: SIBAR), the Effort-Reward Imbalance at Work Questionnaire (German Abbrev.: ERI) and the Occupational Stress and Coping Inventory (German Abbrev.: AVEM), which are validated self-rating instruments frequently used in the rehabilitation setting to identify patients with work-related problems. The SIBAR [39] provides information on potential risk factors for early retirement: the intention to apply for a disability pension (answers were “yes” vs. “no”), patients’ self-perceived work ability (answers were “not being able to work (<3 h/day)”, “limited work ability (3-6 hours/day)” and “full work ability (>6 h/day”), patients’ self-perceived capacity to return to the former job and related working tasks (answers were “definitely yes”, “probably yes”, “uncertain”, “probably no”, “definitely no”), duration of sick leave in the year preceding the rehabilitation program (answers were “no sick leave”, “0–5 weeks”, “6–25 weeks” and “26 weeks and more”), and feelings of occupational stress (answers were dichotomized into  “yes” (=“very stressed”) vs. “no” (=“somewhat stressed” to “job is very fullfilling”)). The ERI was applied to measure the amount of effort spent at work and the reward gained in return. Subscale means for effort and reward range from 0 to 5, with higher values reflecting either higher effort or reward. The ERI-ratio can be calculated to assess the individual’s effort-reward imbalance, which is indicated by a score of ≥1 [40, 41]. The AVEM assesses work behavior in three domains relevant for professional demands and health (work commitment, resistance to stress, emotions). Individuals can be categorized into one of four work-related behavior patterns and coping styles: healthy-ambitious (Type G), unambitious (Type S), excessively ambitious (Risk Type A) and resigned (Risk Type B) [42]. Questionnaires specifically developed for use in this study are provided as Additional file 1).

### Recruitment procedures and nonresponder analysis

#### Recruitment of survivors

**Fig. 1:**
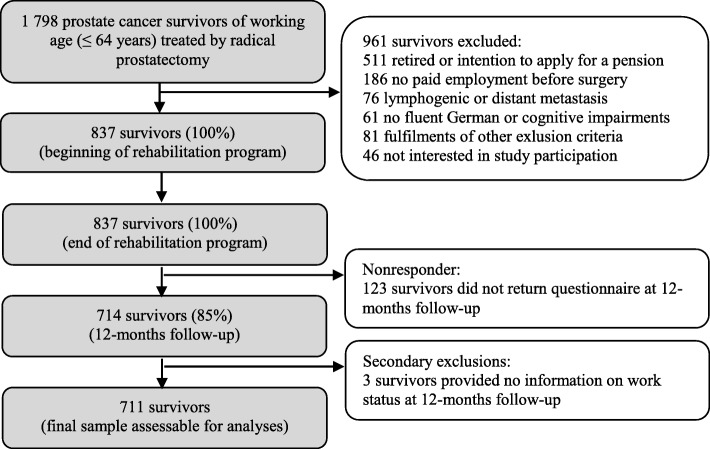
Flow chart of questionnaire responses

During the study period, 1798 survivors of working age who had been treated for localized prostate cancer by radical prostatectomy were admitted to the participating rehabilitation clinics. Overall, 837 survivors met the inclusion criteria and responded to the first two questionnaires at the beginning and the end of the rehabilitation program. The response rate at 12-months follow-up was 85% (714 survivors). As three survivors did not report their work status at follow-up, 711 cases were assessable for the presented analyses (Fig. 1).

#### Nonresponder analyses

Differences between responders and nonresponders at 12-months follow-up were assessed regarding socio-demographic, disease-specific and psychological characteristics. At the beginning of the rehabilitation program, responders were significantly older (57 vs. 56 years) and more frequently married (84 vs. 75%) than nonresponders. However, a logistic regression analysis showed that those variables could only explain a small part of the response variation (Nagelkerkes R^2^: 0.047).

### Statistical analysis

We performed descriptive analyses to examine study population characteristics and to assess the RTW rate and time until RTW at 12-months follow-up. For comparison of baseline characteristics of the survivor groups (returned vs. not returned to work), we conducted univariate analyses using chi-square-tests and two-sample t-tests. Associations between potential predictor variables and RTW outcomes at follow-up were analyzed using multivariate logistic regression models with no RTW and late RTW (≥ 8 weeks) being the dependent variables. Survivors who had returned to work and those with early RTW (< 8 weeks) were classified as reference groups, respectively. Therefore, potential predictors - including all variables that revealed significant group differences in the univariate analyses - were tested for correlation and multicollinearity (spearman’s coefficient rho ≥0.6, tolerance values ≤0.6). Based on the approach of theoretical and statistical pre-selection of variables, all remaining potential predictors were entered simultaneously into the regression analyses (method: enter). Missing data was handled by list-wise deletion and the strengths of associations were expressed as odds ratios (OR) with 95% confidence intervals (CI). All significance tests were two-tailed using a significance level of α < .05. Analyses were performed using SPSS software version 18.0.

## Results

### Study population characteristics

Of 711 survivors, 84% were married, 47% low-educated, and the mean age was 57 years (range: 40–64). On average, survivors had been diagnosed with prostate cancer approximately 3 months prior to the program, with UICC tumor stage II being most prevalent. Fifty-two percent had been treated with open radical prostatectomy and 48% with laparoscopic or robotic approaches (Table 1).

**Table 1:** Characteristics of the responders at the beginning of the cancer rehabilitation program (*N* = 711)

	Whole sample
	*N* = 711
Age, M (SD)	57.0 (4.4)
Age groups, *n* (%)	
Up to 60 years	555 (66.3)
60 years and older	282 (33.7)
Family status, *n* (%)	
Married	591 (83.8)
Single	44 (6.2)
Separated, divorced or widowed	70 (9.9)
Educational level, *n* (%)	
Up to 9 years	324 (46.9)
10 years	156 (22.6)
12–13 years	211 (30.5)
Work status, n (%)	
Full-time	663 (95.9)
Part-time	28 (4.1)
Type of occupation, *n* (%)	
Blue-collar job	247 (35.1)
White-collar job	352 (50.1)
Self-employed or public servant	104 (14.8)
Monthly household net income, *n* (%)	
< 2000 €	136 (20.0)
2000- < 3000 €	237 (34.9)
3000- < 4000 €	187 (27.5)
4000 € or more	119 (17.5)
Tumor stage at diagnosis (UICC)^a^, *n* (%)	
Stage I	82 (11.5)
Stage II	480 (67.6)
Stage III	148 (20.8)
Time since diagnosis (in months)^b^, M (SD)	2.8 (5.0)
Number of comorbid conditions	
None	279 (39.2)
1	254 (35.7)
2 or more	178 (25.0)
Surgical procedure (radical prostatectomy), *n* (%)	
Open (retropubic or perineal)	369 (51.9)
Laparoscopic	95 (13.4)
Robot-assisted (DaVinci)	247 (34.7)

^a^*UICC* International Union against Cancer

^b^Prostate cancer diagnosis via punch biopsy

### RTW rate at 12-months follow-up

Sixhundred-eighteen survivors (87%) had returned to work*.* Reasons for not working were being on sick leave in 23 cases, being unemployed in 21, receiving retirement pension in 30, and disability pension in 19 (data not shown). Univariate analyses showed significant group differences between survivors who had vs. had not returned to work regarding socio-demographic and disease-related characteristics, psychosocial well-being and work-related factors, with the latter being the most affected dimension (Tables 2 and 3).

**Table 2:** Socio-demographic and disease-specific characteristics of prostate cancer survivors at the beginning of the cancer rehabilitation program with regard to work status at 12-months follow-up (*N* = 711)

	Not returned to work 12 months after the end of the rehabilitation program				Returned to work 12 months after the end of the rehabilitation program				
	*N* = 93				*N* = 618				
	n	%	M	SD	n	%	M	SD	*p*-value
Socio-demographic characteristics									
Age	93		59.7	3.2	618		56.9	4.4	**<.001** ^a^
Family status									
Married	77	83.7			514	83.8			.970^b^
Other	15	16.3			99	16.2			
Educational level									
Up to 9 years	45	50.6			279	46.3			.442^b^
10 years	22	24.7			134	22.3			
12–13 years	22	24.7			189	31.4			
Occupational status									
Blue -collar job	34	37.4			213	34.8			.369^b^
White -collar job	40	44.0			312	51.0			
Self-employed or public servant	17	18.7			87	14.2			
Monthly household net income									
< 2000 €	24	27.9			95	16.0			**.024** ^b^
2000- < 4000 €	48	55.8			376	63.4			
4000 € or more	14	16.3			122	20.6			
Disease-specific characteristics									
Surgical procedure									
Open (retropubic or perineal)	48	51.6			321	51.9			.953^b^
Laparoscopic or robot-assisted (DaVinci)	45	48.4			297	48.1			
UICC tumor stage^c^									
Stage I or II	61	65.6			501	81.2			**.001** ^b^
Stage III	32	34.4			116	18.8			
Time since diagnosis (via punch biopsy) in months	93		3.0	6.6	618		2.8	4.7	.665^a^
Karnofsky performance status (0–100%)	93		78.5	7.7	618		79.3	8.8	.412^a^
Extent of urinary incontinence									
°0- no leakage	7	7.5			90	14.6			.179^b^
°1- only in the afternoon	21	22.6			158	25.6			
°2- already before noon	23	24.7			140	22.7			
°3- also at night	42	45.2			228	37.0			
Number of comorbid conditions									
None	27	29.0			252	40.8			.053^b^
1	35	37.6			219	35.4			
2 or more	31	33.3			147	23.8			
Setting of the cancer rehabilitation program									
Inpatient	82	88.2			535	86.6			.671^b^
Outpatient	11	11.8			83	13.4			

Abbreviations *M* mean, *SD* Standard deviation, *p*-value, probability of type I error

Significant *p*-values are marked in bold

^a^t-test, two-tailed

^b^chi-square-test

^c^*UICC* International Union against Cancer

**Table 3:** Psychosocial and work-related factors of prostate cancer survivors at the beginning of the rehabilitation program with regard to work status at 12-months follow-up (*N* = 711)

	Not returned to work 12 months after the end of the rehabilitation program				Returned to work 12 months after the end of the rehabilitation program				
	*N* = 93				*N* = 618				
	n	%	M	SD	n	%	M	SD	*p*-value
Psychosocial well-being, function and symptoms									
Anxiety and Depression (HADS)									
Anxiety	93		6.2	4.3	616		5.6	3.8	.149^a^
Depression	93		5.4	4.0	617		4.8	3.4	.146^a^
Quality of Life – functioning (EORTC QLQ-C30)^b^									
Global health status/ quality of life	93		48.1	22.4	618		53.1	20.6	**.032** ^a^
Physical functioning	93		68.2	20.9	617		72.9	19.0	**.031** ^a^
Role functioning	93		37.1	31.4	615		40.6	33.7	.350^a^
Emotional functioning	93		61.0	27.8	615		64.0	24.9	.283^a^
Cognitive functioning	93		77.1	26.7	616		78.6	22.8	.547^a^
Social functioning	93		50.7	30.0	618		56.1	27.7	.083^a^
Quality of life – symptoms (EORTC QLQ-PR25)^c^									
Urinary symptoms	93		48.2	19.5	615		45.7	20.0	.265^a^
Bowel symptoms	92		10.4	13.1	614		8.3	11.4	.141^a^
Hormonal treatment-related symptoms	92		16.0	13.3	617		14.0	12.2	.143^a^
Bother due to use of incontinence aid	73		47.0	35.9	452		42.1	33.5	.249^a^
Work-related issues and behaviors									
Work-related behavior pattern (AVEM)									
Healthy ambitious- Type G	25	26.9			156	25.2			.092^d^
Unambitious- Type S	37	39.8			196	31.7			
Excessively unambitious- Risk Type A	14	15.1			112	18.1			
Resigned- Risk Type B	16	17.2			102	16.5			
Unclear	1	1.1			52	8.4			
Work-related issues (SIBAR)									
Self-perceived work ability		92			615				
Not able to work (< 3 h/day)		38	41.3		124	20.2			**<.001** ^d^
Limited ability (3–6 h/day)		49	53.3		415	67.5			
Full ability (> 6 h/day)		5	5.4		76	12.4			
Sick leave in the 12 months preceding rehabilitation									
None or up to 5 weeks	55	60.4			499	82.3			**<.001** ^d^
6 weeks or more	36	39.6			107	17.7			
Intention to apply for a disability pension (yes)	39	43.8			124	20.6			**<.001** ^d^
Occupational stress (yes)	22	24.2			76	12.4			**.002** ^d^
Self-perceived capacity to return to the former job and related working tasks									
Probably or definitely yes	55	59.1			532	86.6			**<.001** ^d^
Uncertain	23	24.7			67	10.9			
Probably or definitely no	15	16.1			15	2.4			
Effort-reward imbalance (ERI)									
Effort^e^	89		16.5	5.1	614		15.5	4.4	.094^a^
Reward	85		46.4	7.6	591		48.2	6.8	**.022** ^a^
Effort-reward imbalance (cut off ≥1)	12	14.1			48	8.1			.071^d^

Abbreviations *M* mean, *SD* Standard deviation, *p*-value probability of type I error

Significant *p*-values are marked in bold

^a^t-test, two-tailed

^b^scale 0–100 (100 ≅ maximum level of functioning), symptom scales not included in the presented analyses

^c^scale 0–100 (100 ≅ maximum symptom burden), functioning scales not included in the presented analyses

^d^chi-square-test

^e^analyses based on the six-item version

### Time until RTW following the cancer rehabilitation program

**Fig. 2:**
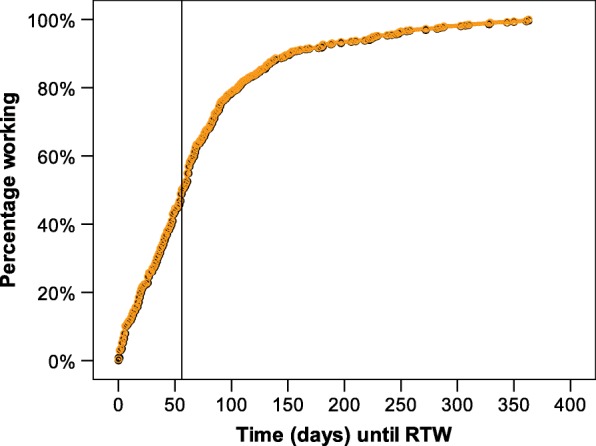
Return to work (RTW; in days) of prostate cancer survivors within the 12 months following the cancer rehabilitation program (*N* = 549)

Among 618 survivors who had returned to work, the exact date of RTW was not available in 69, leaving 549 for the analysis of time until RTW. Survivors returned to work with a median time of 56 days (mean 73.7, standard deviation 70.6, range: 0–365). Figure 2 depicts descriptive data on the days patients needed to return to work after the end of rehabilitation (100% = 549 survivors having returned to work within 1 year following the program).

### Predictors of not having returned to work at 12-months follow-up

In the multivariate regression model, older age (OR 1.247), UICC tumor stage III (OR 2.268), sick leave of 6 weeks and more (in the year preceding the rehabilitation program; OR 2.981), patients’ self-perceived (baseline) inability to work (OR 3.671), lacking capacity to return to the former job and related working tasks (3.162) and intention to apply for a disability pension (OR 2.214) increased the likelihood for not having returned to work at 12-months follow-up (Table 4). The regression model explained 28% of the total variance (Nagelkerke’s R^2^: 0.283).

**Table 4:** Results of the multivariate regression models of having returned to work and late return to work at 12-months follow-up

	Multivariate regression analyses									
	Not returned to work 12 months after the end of the rehabilitation program *N* = 617^a^					Late return to work (≥ 8 weeks) following the cancer rehabilitation program *N* = 491^b^				
	β	SE	*p*-value^c^	OR	95% CI	β	SE	*p*- value^c^	OR	95% CI
Age	.221	.046	**<.001**	1.247	1.139–1.366	.018	.023	.452	1.018	.972–1.066
Monthly household net income										
4000 € and more	Ref.					Ref.				
2000 - < 4000 €	−.134	.379	.724	.875	.416–1.837	.439	.271	.106	1.552	.911–2.641
< 2000 €	.198	.467	.671	1.219	.488–3.043	.604	.373	.105	1.830	.881–3.801
Tumor stage (UICC^d^)										
Stage I or II	Ref.					Ref.				
Stage III	.819	.315	**.009**	2.268	1.223–4.028	1.080	.259	**<.001**	2.946	1.773–4.894
Global health status/Quality of life (EORTC QLQ-C30)	−.001	.008	.893	.999	.983–1.015	−.002	.006	.780	.998	.987–1.010
Physical functioning (EORTC QLQ-C30)	−.003	.462	.691	.997	.980–1.013	−.009	.006	.172	.991	.979–1.004
Sick leave in the 12 months preceding rehabilitation (SIBAR)										
None or up to 5 weeks	Ref.					Ref.				
6 weeks or more	1.092	.308	**<.001**	2.981	1.629–5.456	.249	.266	.348	1.283	.762–2.160
Self-perceived work ability (SIBAR)										
Full ability (> 6 h/day)	Ref.					Ref.				
Limited ability (3–6 h/day)	.305	.526	.562	1.357	.484–3.809	.768	.345	**.026**	2.154	1.095–4.283
Not able to work (< 3 h/day)	1.300	.589	**.027**	3.671	1.156–11.653	1.505	.421	**<.001**	4.502	1.971–10.284
Self-perceived capacity to return to the former job (SIBAR)										
Probably or definitely yes	Ref.					Ref.				
Uncertain	.504	.339	.206	1.656	.758–3.618	1.056	.398	**.008**	2.876	1.319–6.271
Probably or definitely no	1.151	.580	**.047**	3.162	1.014–9.861	.896	.637	.160	2.450	.072–8.545
Intention to apply for a disability pension (SIBAR)										
No	Ref.					Ref.				
Yes	.795	.312	**.011**	2.214	1.200–4.083	.326	.256	.219	1.385	.824–2.328
Occupational stress (SIBAR)										
No	Ref.					Ref.				
Yes	−.608	.462	.189	.545	.220–1.347	.249	.356	.442	1.315	.654–2.264
Reward (ERI)	.001	.023	.979	1.001	.957–1.046	−.009	.017	.593	.991	.959–1.024

Abbreviations *ß* unstandardized regression coefficient, *SE* Standard error, *p*-value Probability of type I error, *OR* odds ratio for independent variables, *CI* 95% confidence interval

Significant *p*-values are marked in bold

^a^Reference group: Having returned to work; due to missing values within the predictor variables, 617 out of 711 survivors were included into the final regression model; tolerance values between .675–.978

^b^Reference group: early return to work (< 8 weeks); due to missing values within the predictor variables, 491 out of 549 survivors were included into the final regression model; tolerance values between .700–.977

^c^Wald Test

^d^*UICC* International Union against Cancer

### Predictors of late return to work (≥ 8 weeks) following the cancer rehabilitation program

In the multivariate regression model, UICC tumor stage III (OR 2.946), and patients’ self-perceived (baseline) limited work ability (OR 2.154) and not being able to work (OR 4.502) as well as uncertainty about the capacity to return to the former job and related working tasks (OR 2.876) were significant predictors for late RTW (Table 4). The regression model explained 22% of the total variance (Nagelkerke’s R^2^: 0.215).

## Discussion

This prospective multicentre-study analyzed the RTW rate and time until RTW in a cohort of 711 prostate cancer survivors 12 months after having attended a cancer rehabilitation program. Previous international studies demonstrated RTW rates of cancer survivors ranging from 24 to 94% 1 year post diagnosis [5]. Regarding the population of prostate cancer patients, international studies suggest relatively high RTW rates [8, 9, 24]. For example, among working age prostate cancer patients who had received radiotherapy, 75% were reported to be available for work 1 year after treatment [43]. In our study, 87% of survivors had returned to work 12 months after the end of the rehabilitation program. Thus, the RTW rate was higher compared to results from two other studies conducted in the German cancer rehabilitation setting. Both studies analyzed mixed samples (both genders and different cancer types) and revealed RTW rates of 79% [44] and 76% [45] by 1 year after the rehabilitation program. However, such comparison of RTW rates has to take into account that in our study, only cancer survivors who were active in the workforce before radical prostatectomy were included.

Overall, prostate cancer patients seem to return to work faster when compared with patient groups diagnosed with other cancer types [46]. In our study, median time until RTW was 56 days, while other studies reported a five-week median time until RTW in urologic (specifically prostate) cancer patients from the U.K. [46] and a median sickness absence of 20 days in U.S. prostate cancer patients [11]. In a study with Norwegian prostate cancer patients who were employed before radical prostatectomy, 51% had returned to work within 6 weeks and 73% within 9–10 weeks post-operative [47]. Comparing robot-assisted laparoscopic to open radical prostatectomy among prostate cancer patients, studies demonstrated a shorter time until RTW (35 vs. 48 days) in Swedish patients [48] and a shorter median sick leave (11 vs. 49 days) in Swedish/Danish patients [49]. In our study, approximately half of survivors had been treated with open prostatectomy. Thus, the median amount of 56 days needed to RTW seems to support findings of these studies.

However, comparability of our data with international studies is limited due to heterogeneous healthcare and/or social systems as well as the uniqueness of the German rehabilitation system.

Further, we investigated baseline risk factors for not having returned to work at 12-months follow-up and late RTW. Although univariate analyses showed global quality of life and physical functioning to be significantly lower in patients who had not returned to work, those aspects were not relevant in the multivariate analyses. None of the physical symptoms or disease-related lasting effects seemed to have an impact, while reviews focusing on RTW after cancer suggest fatigue and other physical symptoms to be important predictors for RTW outcomes [5, 32]. In prostate cancer patients, constipation was found to predict longer RTW [46] and pre-operative physical health-related quality of life was predictive for declined work status 3 months after radical prostatectomy [47].

Interestingly, the survivors’ age was of no significant impact regarding time until RTW. In a study with employed Norwegian prostate cancer patients after radical prostatectomy, age was found to be a risk factor for prolonged sick leave [47]. In our study, as opposed to others [50], the upper age limit was set at 64 years, as the age limit for old age pension in Germany has been raised to up to 67 years and early retirement can cause financial losses or predicaments. Thus, RTW and work-related issues are relevant even in this age group and facilitating RTW within medical rehabilitation programs has been an important point of interest for the German Pension Insurance Agency, reflected by the slogan “rehabilitation before retirement” [25, 27].

Consistent with previous studies [44–46], the results of the multivariate logistic regression analyses demonstrate that survivor’s perceptions in relation to work impact the RTW process.

In our study, patients’ baseline perceptions of no and/or limited work ability as well as uncertain or no capacity to return to the former job were strong prognostic factors for both not having returned to work at 12-months follow-up and late RTW (≥ 8 weeks). While personal and disease-specific determinants cannot be changed, perceptions about future work might be modifiable during cancer rehabilitation programs. Assessing and responding to adverse perceptions are important goals of occupation-directed interventions in cancer patients [51, 52]. Helping patients to prepare for RTW and to modify maladaptive perceptions through psycho-educational interventions, counseling and advice are core functions of German cancer rehabilitation programs. As was shown in a recent study, an “add-on” structured occupationally oriented rehabilitation program led to better patient ratings of subjective work ability than care as usual [53].

Our results suggest to screen prostate cancer survivors’ perceptions in relation to work in order to promote RTW rates and early occupational reintegration. Prospectively, reliable screenings could improve the early and differentiated referral of at-risk survivors to intensified occupational support, both during rehabilitation programs and beyond. In view of evidence-based screening strategies, further research is needed to investigate factors that might increase the probability of not returning to work or prolonged RTW trajectories. Further, in order to organize such support, reasons of survivors’ negative perceptions, for example feeling incapable to return to work or their intention to apply for a disability pension, need to be clarified.

Our study has strengths and limitations. In this large-scale longitudinal study, we consecutively collected data from a well-defined population of employed prostate cancer survivors after radical prostatectomy who enrolled in multidisciplinary cancer rehabilitation programs. Reasons for excluding patients from study participation were thoroughly documented. We were able to recruit a large sample size in four specialized German rehabilitation clinics, with a response rate of over 80% at 12-months follow-up. Another strength of our study was that we used patient-reported outcomes regarding survivors’ work status, psychosocial well-being and work-related factors.

Yet, it is notable, that the results of this study are subject to certain limitations. Among those, the most important was generizability of results. First, our study did not include a control group of rehabilitation non-participants. We cannot assess possible selection bias regarding rehabilitation participants and if RTW outcomes differ between participants and non-participants. Therefore, our results cannot be generalized to non-participants.

Second, half of the patients were treated by open radical prostatectomy, resulting in a strong representation of the respective surgical procedure and associated side effects. Since minimally invasive surgical approaches offer potentially shorter recovery times [54], generizability of the results should be applied with awareness for possible bias in the outcome parameter of time until RTW as well as psychosocial and work-related predictor variables.

Third, early retirement or having applied for a disability pension were used as exclusion criteria (511 patients of the total sample affected), and this might have impacted the results. However, we did not have information on reasons for early retirement or having applied for a disability pension in these patients. Generally, prostate cancer is a disease of older age [13], which may lead to higher early retirement rates in this patient population and may be an aggravating factor in studying RTW as an outcome measure.

Further, our regression model explained a rather moderate ratio of the overall variance in the dependent variable. The regression analyses were aimed at testing predicted factors for not having returned to work and late RTW based on a model of cancer and work proposed by Feuerstein et al. [32]. We acknowledge that there are other important predictors that have a close relationship with RTW and time until RTW that are not considered, leading to the lower amount of variance explained in the regression. However, our study shows that the predicted factors have a significant impact on both outcomes.

Based on the multicentre design, consecutive recruitment strategy, systematic documentation of nonresponders, a high response rate at all times of measurement, and theoretically and statistically derived predictor variables, we consider our results to be valid for employed prostate cancer survivors who participated in a cancer rehabilitation program.

## Conclusions

Next to recovery from physical impairments, the purpose of cancer rehabilitation programs is to improve the individuals’ psychological and social functioning, including the ability to return to work. Our findings highlight that RTW in prostate cancer survivors who were active in the working force pre-surgery and attended a cancer rehabilitation program is a realistic goal. Those, who are not able to return to work or who return late seem to be a subgroup of survivors. Results underline the importance of prostate cancer survivor’s perceptions in relation to work and indicate the need for reliable screening procedures to early identify survivors at risk for adverse RTW outcomes. Those may help to direct the rehabilitation process with regard to intensified occupational support.

## Additional file

Additional file 1: Questionnaires developed specifically for use in this study. (DOCX 16 kb)

## Abbreviations

AVEM: Occupational Stress and Coping Inventory (German Abbreviation)
CI: Confidence interval
EORTC: European Organization for Research and Treatment of Cancer
ERI: Effort-Reward Imbalance at Work Questionnaire (German Abbreviation)
HADS: Hospital Anxiety and Depression Scale
ICF: International Classification of Functioning, Disability and Health
OR: Odds ratio
RTW: Return to work
SIBAR: Screening Instrument Work and Occupation (German Abbreviation)
UICC: International Union Against Cancer
