# Supplementary material for: Return to work in prostate cancer survivors – findings from a prospective study on occupational reintegration following a cancer rehabilitation program
Source: BMC Cancer. 2018 Jul 20;18:751. doi: 10.1186/s12885-018-4614-0 (PMC6053748; doi:10.1186/s12885-018-4614-0)
Supplement: Supplementary file 1 — Questionnaires developed specifically for use in this study. (DOCX 16 kb) [file 12885_2018_4614_MOESM1_ESM.docx]

**QUESTIONNAIRES DEVELOPED SPECIFICALLY FOR USE IN THIS STUDY**

**DATA FROM PATIENT SELF-REPORT QUESTIONNAIRES**

**T1 – beginning of the rehabilitation program: Socio-demographic variables used for description of patient characteristics and /or potential predictor variables**

**Date of birth (age was calculated by linkage of date of birth and date of admission to the rehabilitation clinic retrieved from medical records):**

__ __.__ __ __ __ (mm/yyyy)

**Marital status:**

Single (never married)

Married (living together with partner)

Married (separated)

Divorced

Widowed

**T3 – 12-months follow-up: Variables on RTW outcomes**

**Current work status:**

Are you currently employed? Please answer this question even if you are currently on sick leave.

Yes, full-time

Yes, part-time, working ____ hours per week

No, unemployed since __ __.__ __ __ __ (mm/yyyy)

No, undergoing retraining

No, disability pension since __ __.__ __ __ __ (mm/yyyy)

No, old-age pension (retirement)

Other: _____________________

**Date of return to work (time since return to work was calculated by linkage of date of return and date of discharge from the rehabilitation clinic retrieved from medical records):**

Please provide the exact date on which you have returned to work (meaning any payed employment after the end of the rehabilitation program).

Date of return to work: __ __.__ __.__ __ __ __ (dd/mm/yyyy)

I have not returned to work yet.

**DATA PROVIDED BY PHYSICIANS OR RETRIEVED FROM MEDICAL RECORDS**

**T1 – beginning of the rehabilitation program: Medical information on patients used for description of sample characteristics and / or as potential predictor variables:**

**TNM classification for prostate cancer:**

T (pathological): ____

N (pathological): ____ ( ____ ____ )

M (clinical): ____

**Date of first diagnosis via punch biopsy (time since diagnosis was calculated by date of first diagnosis and date of admission to the rehabilitation clinic retrieved from medical records):**

__ __.__ __ __ __ (mm/yyyy)

**Surgical procedure:**

Retropubic

Perineal

Laparoscopic

Robot-assisted (DaVinci)

**Setting of the cancer rehabilitation program:**

Outpatient

Inpatient

**Extent of urinary incontinence:**

°0: no leakage

°1: only in the afternoon

°2: already before noon

°3: also at night

**Comorbid conditions which are or must be submitted to a treatment (number of comorbid conditions was then calculated from the data obtained):**

Cardiovascular: ICD-10: _____________ ICD-10: _____________ ICD-10: _____________

Diabetes: ICD-10: _____________ ICD-10: _____________ ICD-10: _____________

Neurologic: ICD-10: _____________ ICD-10: _____________ ICD-10: _____________

Orthopaedic: ICD-10: _____________ ICD-10: _____________ ICD-10: _____________

Psychiatric: ICD-10: _____________ ICD-10: _____________ ICD-10: _____________

Others: ___________________________: ICD-10: _____________

Others: ___________________________: ICD-10: _____________

Others: ___________________________ : ICD-10: _____________

**T1- beginning of the rehabilitation program / T2- end of the rehabilitation program: Dates used for calculation of timespans**

Dates of admission to the rehabilitation program (dd/mm/yyyy) and date of discharge (dd/mm/yyyy) were derived from reports which are completed for each patient leaving the rehabilitation clinic. The standard form of the German Pension Insurance Agency used for these reports can be downloaded via the following link: https://www.deutsche-rentenversicherung.de/Allgemein/de/Inhalt/5_Services/04_formulare_und_antraege/03_reha_einrichtungen/_DRV_Paket_Rehaeinrichtungen_Aerztlicher_Entlassungsbericht.html
